# Isoprenoid alcohols utilization by malaria parasites

**DOI:** 10.3389/fchem.2022.1035548

**Published:** 2022-12-01

**Authors:** Ignasi Bofill Verdaguer, Rodrigo A. C. Sussmann, Verônica Feijoli Santiago, Giuseppe Palmisano, Gabriel Cândido Moura, Juliana Tonini Mesquita, Lydia Fumiko Yamaguchi, Massuo Jorge Kato, Alejandro Miguel Katzin, Marcell Crispim

**Affiliations:** ^1^ Department of Parasitology, Institute of Biomedical Sciences of the University of São Paulo, São Paulo, Brazil; ^2^ Center for Environmental Sciences, Institute of Humanities, Arts and Sciences, Federal University of Southern Bahia, Bahia, Brazil; ^3^ Department of Fundamental Chemistry, Institute of Chemistry, University of São Paulo, São Paulo, Brazil

**Keywords:** dolichol, phytol, geranylgeraniol, farnesol, *P. falciparum*, fosmidomycin, malaria, isoprenoid

## Abstract

*Plasmodium falciparum* is the etiological agent of human malaria, one of the most widespread diseases in tropical and subtropical regions. Drug resistance is one of the biggest problems in controlling the disease, which leads to the need to discover new antimalarial compounds. One of the most promissory drugs purposed is fosmidomycin, an inhibitor of the biosynthesis of isoprene units by the methylerythritol 4-phosphate (MEP) pathway, which in some cases failed in clinical studies. Once formed, isoprene units are condensed to form longer structures such as farnesyl and geranylgeranyl pyrophosphate, which are necessary for Heme O and A formation, ubiquinone, and dolichyl phosphate biosynthesis as well as for protein isoprenylation. Even though the natural substrates of polyprenyl transferases and synthases are polyprenyl pyrophosphates, it was already demonstrated that isoprenoid alcohols (polyprenols) such as farnesol (FOH) and geranylgeraniol (GGOH) can rescue parasites from fosmidomycin. This study better investigated how this rescue phenomenon occurs by performing drug-rescue assays. Similarly, to FOH and GGOH, it was observed that phytol (POH), a 20-carbon plant isoprenoid, as well as unsaponifiable lipid extracts from foods rescue parasites from the antimalarial effect of fosmidomycin. Contrarily, neither dolichols nor nonaprenol rescue parasites from fosmidomycin. Considering this, here we characterized the transport of FOH, GGOH, and POH. Once incorporated, it was observed that these substances are phosphorylated, condensed into longer isoprenoid alcohols, and incorporated into proteins and dolichyl phosphates. Through proteomic and radiolabelling approaches, it was found that prenylated proteins are naturally attached to several isoprenoids, derived from GGOH, dolichol, and POH if exogenously added. Furthermore, the results suggest the presence of at least two promiscuous protein prenyltransferases in the parasite: one enzyme which can use FPP among other unidentified substrates and another enzyme that can use GGPP, phytyl pyrophosphate (PPP), and dolichols, among other substrates not identified here. Thus, further evidence was obtained for dolichols and other isoprenoid products attached to proteins. This study helps to better understand the apicoplast-targeting antimalarial mechanism of action and a novel post-translational modification of proteins in *P. falciparum*.

## Introduction

*Plasmodium falciparum* is the etiological agent of human malaria, a disease spread in tropical and subtropical regions of Africa, Southeast Asia, and America (World Health Organization, 2021). Drug resistance is one of the biggest problems in controlling the disease, which has led to the need to discover new antimalarial compounds and to better understand the biological rationale under the drug-resistance mechanisms (Boland and World Health Organization, 2001). In this context, biological mechanisms and cellular structures present in the parasite and absent in humans represent a great attraction for developing new drugs and, therefore, whenever discoveries become an intense research target.

One of the most studied organelles in *Plasmodium* is the apicoplast, a non-photosynthetic plastid absents in humans (Seeber and Soldati-Favre, 2010). This organelle has a metabolic pathway for the production of isoprenoids that is not present in humans: the methylerythritol 4-phosphate (MEP) pathway, essential for *Plasmodium* (Cassera et al., 2004; Sparr et al., 2013).

**FIGURE 1:**
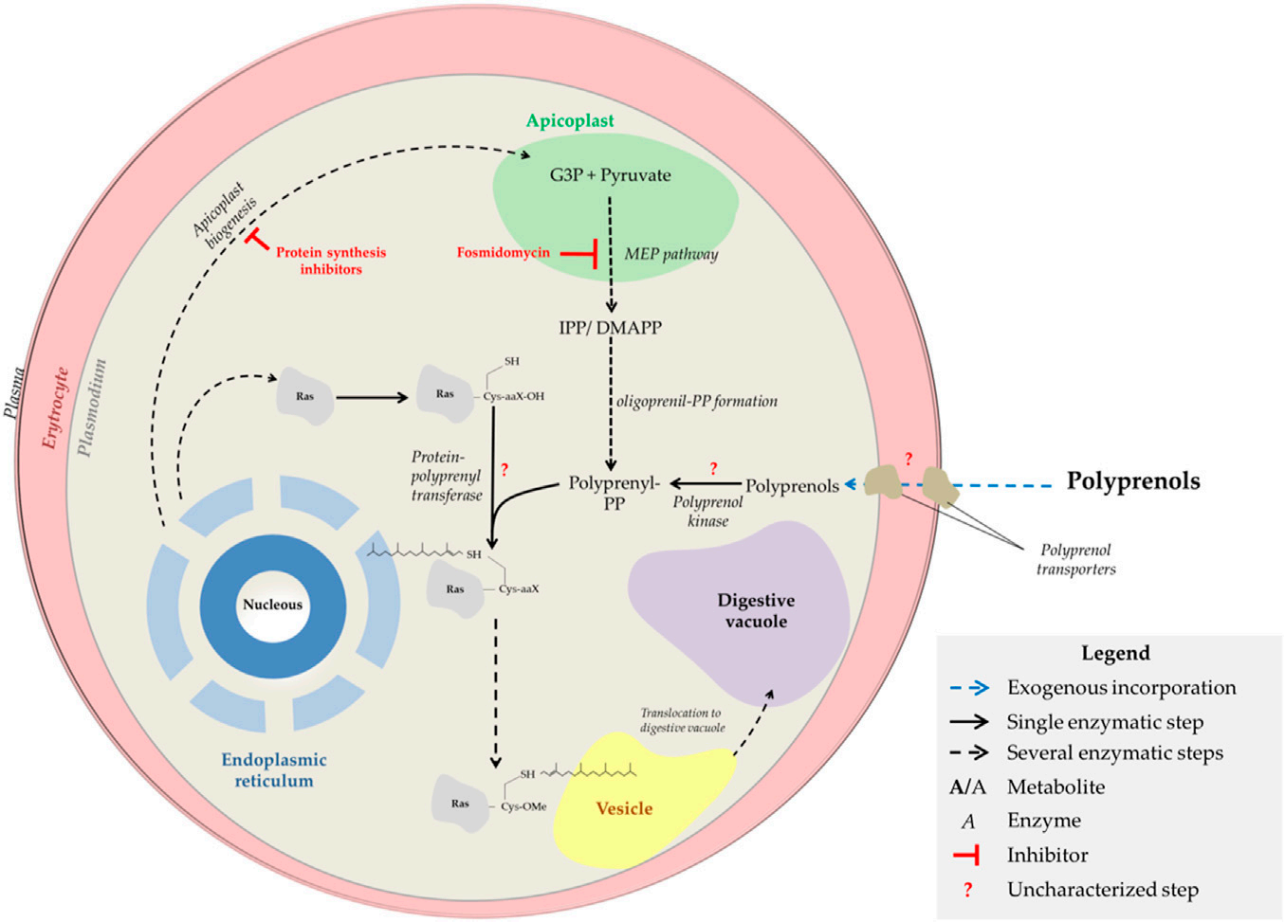
Isoprenoid sources and distribution in malaria parasites. The scheme indicates isoprenoid sources and distribution as well as fosmidomycin and ribosomal inhibitors targets in malaria parasites.

The MEP pathway leads to the formation of isoprene units, isopentenyl pyrophosphate (IPP), and its isomer dimethylallyl pyrophosphate (DMAPP). Longer isoprenoid pyrophosphates are formed by polyprenyl synthases, which condense consecutively several isoprene units (Okada et al., 2022; Tonhosolo et al., 2005). The simplest oligoprenyl pyrophosphates are geranyl pyrophosphate (10 carbon), farnesyl pyrophosphate (FPP, 15 carbon), and geranylgeranyl pyrophosphate (GGPP, 20 carbon) which are directly used for farnesylation and geranylgeranylation of proteins (Figure 1). Reports in the literature indicate that FPP can also be used for the formation of Heme-O (Simão-Gurge et al., 2019). The parasite also should produce longer prenyl pyrophosphates such as octaprenyl and nonaprenyl pyrophosphate (solanesyl pyrophosphate) necessary for the biosynthesis of ubiquinone, *cis*-polyprenols, and dolichols of 11, 12, and 15-19 isoprene units (Okada et al., 2022; D'Alexandri et al., 2006; Zimbres et al., 2020; Verdaguer et al., 2021; Verdaguer et al., 2019). Finally, some authors previously suggested that the parasite can use phytol (POH) to synthesize vitamin E (Sussmann et al., 2011). POH ((2E,7R,11R)-3,7,11,15-Tetramethylhexadec-2-en-1-ol) is an acyclic diterpene produced through the hydrogenation of GGPP (Hansen, 1980; Proteau, 1998; Sussmann et al., 2022). In the parasite, Sussmann et al. (Sussmann et al., 2011) observed the incorporation of exogenous [1-(n)-^3^H]-POH into prenylquinones. However, is not known if the parasite can produce POH or obtain it from the exogenous environment.

Dolichols play a role as sugar anchors for the glycosylation of proteins and other molecules (Parodi and Leloir, 1979; de Macedo et al., 2010). *P. falciparum* proteins can be modified with *N*- (Gowda and Davidson, 1999; Cova et al., 2015; Goddard-Borger and Boddey, 2018; Goerdeler et al., 2021; Fenollar et al., 2022) and *O*-glycans (Dieckmann-Schuppert et al., 1993; Bandini et al., 2019), single fucose (Lopaticki et al., 2017) or mannose (López-Gutiérrez et al., 2019; Srivastava et al., 2022), and glycosylphosphatidylinositol anchor modification of proteins (Gowda and Davidson, 1999). Besides protein glycosylation, dolichols are also directly involved as substrates for protein prenylation (e.g. Ras and Rap proteins). Protein dolichylation in the malaria parasite was observed by D'Alexandri et al., in 2006 (D'Alexandri et al., 2006). Dolichols were also released through a iodomethane treatment of proteins and thus it’s like this kind of modification of proteins occurs through a thioether bond between the lipid and the target protein. D'Alexandri et al., confirmed by mass spectrometry techniques. The authors found that dolichol of 11 isoprene units is bound to 21-28-kDa protein clusters from trophozoite and schizont stages. Furthermore, the analysis of the proteolytic digestion products from parasite proteins labelled with [^35^S] cysteine and [^3^H] FPP showed that the attachment of dolichols to protein is a post-translational event and probably occurs *via* a covalent bond to cysteine residues (D'Alexandri et al., 2006). To our knowledge, this post-translational modification was observed only in malaria parasites and once in human colon carcinoma cells (Hjertman et al., 1997). Still, no further studies about protein dolichylation in *Plasmodium* or any other organism have been published and remains unknown if protein dolichylation occurs at the CAAX domains as typical prenylated proteins.

Since the isoprenoid biosynthesis pathway in *Plasmodium* proved to be an interesting target, the investigation for pathway-inhibiting molecules was intensified. Today it is known that two classes of drugs can inhibit this pathway: Fosmidomycin or its analogues and ribosomal inhibitors (Ex. doxycycline, indolomycin, clindamycin, and azithromycin) (Figure 1) (Dahl and Rosenthal, 2007; Jackson and Dowd, 2012). First, fosmidomycin possesses great inhibitory activity against 1-deoxy-d-xylulose 5-phosphate reductoisomerase (DXR), an MEP-pathway enzyme that catalyzes the conversion of 1-deoxy-d-xylulose 5-phosphate (DXP) into MEP (Figure 1) (Jackson and Dowd, 2012). On the other hand, ribosomal inhibitors interfere with apicoplast biogenesis, promoting organelle fragmentation, and MEP physical disruption; consequently, parasites transmit defective apicoplast to their daughter cells (Dahl and Rosenthal, 2007; Yeh and DeRisi, 2011). For this reason, ribosomal inhibitors affect parasites only from the second intraerythrocytic cycle, i.e. approximately 48 h after treatment begins, a phenotype known as the” delayed death” effect (Yeh and DeRisi, 2011; Kennedy et al., 2019). Parasites treated with fosmidomycin or drugs impairing apicoplast formation can be indefinitely grown *in vitro* if exogenous IPP is added to the culture media (Yeh and DeRisi, 2011), which suggests the MEP pathway be the sole blood-stage essential metabolic pathway in the apicoplast. However, recent findings by Swift and colleagues have demonstrated that other metabolic pathways of the apicoplast are essential and that the apicoplast fragments generated by treatment with ribosomal inhibitors still maintain most of the essential metabolic functions of the original organelle, but not the isoprenoid biosynthesis pathway (Swift et al., 2021). In any case, fosmidomycin clinical trials have failed to cure malaria and ribosomal inhibitors act slowly and possess poor antimalarial activity if not combined with other antimalarial drugs (Dahl and Rosenthal, 2007; Lanaspa et al., 2012; Fernandes et al., 2015; Guggisberg et al., 2016). Therefore, it is necessary to better research on the apicoplast-targeting antimalarial mechanism of action and its effects on the parasite. In this sense, several authors have already investigated the requirements of isoprenoids by the human malaria parasite and characterized the molecular and morphological phenotype of parasites experiencing pharmacological-induced isoprenoid depletion, by interrupting the biogenesis of the apicoplast (Kennedy et al., 2019; Mathews et al., 2019; Tewari et al., 2021). Metabolomics profiling revealed that the decrease in ubiquinone and dolichol biosynthesis is not the primary cause of plasmodial isoprenoid depletion-mediated death, but the disruption of the digestive vacuole function (Kennedy et al., 2019). Interestingly, parasites suffering from this lack of isoprenoid biosynthesis could be rescued by farnesol (FOH) and geranylgeraniol (GGOH) (Yeh and DeRisi, 2011; Kennedy et al., 2019; Mathews et al., 2019). Because of this phenomenon, it was suggested that the loss of isoprenoids impairs protein prenylation and consequently abolishes the function of vesicular trafficking proteins, preventing their membrane attachment and, ultimately, the feeding/nutrition of malaria parasites (Kennedy et al., 2019). FOH and GGOH utilization by parasites indicate the presence of a novel pathway utilizing these metabolites for protein prenylation (Crick et al., 1997). Here it was studied possible sources of isoprenoid alcohols for the parasite, how they are incorporated and metabolized, and how these compounds could be protecting the parasite from apicoplast-targeting antimalarials.

## Materials and methods

### Reagents

Albumax I and RPMI-1640 were purchased from Thermo Fisher Scientific^®^ (Leicestershire, UK). Dolichol and Dolichyl-P 13-21 were purchased from Avanti^®^ (Alabama, United States). [1-^3^H] dolichol 95 from porcine liver (10-20 Ci/mmol; 0.1 mCi/ml), [1-(n)-^3^H], GGOH (14 Ci/mmol; 1 mCi/ml), [1-(n)-^3^H]-POH (20 Ci/mmol; 1 mCi/ml) and [1-(n)-^3^H] FOH (14 Ci/mmol; 1 mCi/ml) were purchased from American Radiolabelled Chemicals^®^ (St. Louis, United States). SYBR Green I^®^ nucleic acid gel stain from Life technologies^®^ (Eugene, OR, United States). American Radiolabelled Chemicals^®^ was also our supplier of non-radiolabelled POH (ARC number: 0286–1 g) together with Merck^®^ (Merck code: 8186050025). Sterile stock solutions were prepared 100 mM for fosmidomycin sodium salt hydrate in water, 50 mM of clindamycin hydrochloride in 70% ethanol, 20 mM for chloroquine diphosphate salt in water, and 200 mM of each non-radiolabelled isoprenoid alcohols in ethanol except dolichols 13-21 which were solubilized in ethanol at 100 mg/ml. Importantly, the purity of both [1-(n)-^3^H]-POH and not radiolabelled POH was guaranteed by NMR and RP-HPLC (see Supplementary Data, Supplementary Figures S1, S2, respectively). The rest of the reagents not commented on here were purchased from Sigma^®^ (St. Louis, Missouri, United States), including fosmidomycin sodium salt hydrate (sigma code F8682).

### *P. falciparum in vitro* culture and synchronization at ring stage

The *P. falciparum* 3D7 isolate was cultured *in vitro* following the Trager and Jensen culture method employing RPMI-1640 medium completed with 0.5% Albumax II into 75 cm^2^ cell culture flasks at 37°C (Trager and Jensen, 1976; Ringwald et al., 1999). The culture medium pH was adjusted to 7.4 and was introduced a gas mixture of 5% CO_2_, 5% O_2_ and 90% N_2_ purchased from Air Products Brasil LTDA^®^ (São Paulo, SP, Brazil). Parasite synchronization at ring stages was performed with a 5% (w/v) D-sorbitol solution as described by Lambros & Vanderberg (Lambros and Vanderberg, 1979). Parasite development was monitored by Giemsa-stained smears microscopy. PCR for *mycoplasma* and optic microscopy were used to avoid culture contamination (Rowe et al., 1998).

### Evaluation of isoprenoid alcohol transport

Synchronous cultures were used at a high parasitemia (minimum 15% parasitemia) in the mature/schizont stage. Infected RBCs were transferred to Falcon tubes and washed once with PBS. The supernatant was removed and the pellet containing only RBCs was distributed into 1.5 ml microtubes, 100 µL per tube. Then tracer solution was added corresponding to 20 µM of the non-radioactive isoprenoid alcohol and 1 μCi/ml of the corresponding radioactive: [1-(n)-^3^H], GGOH, [1-(n)-^3^H]-FOH or [1-(n)-^3^H] POH.

Each tube represented a different incubation time, varying from experiment to experiment, while at the end of the given time 1 ml of ice-cold PBS was added, interrupting the transport process. The samples from the different time points were then washed in ice-cold PBS. To isolate the mature forms from the erythrocytes, the samples were resuspended in 0.03% saponin, homogenized vigorously, and centrifuged. This extraction process was repeated two times. After the last centrifugation, the sample pellets were washed in PBS and resuspended in Ultima Gold^®^ (Perkin Elmer^®^, Waltham, Massachusetts, United States) centrifugation cocktail. The count of detected ionization events per minute (counts per minute, CPM) was monitored on a Beckman LS 5000 TD *β*-counter liquid scintillation spectrometer (Beckman^®^, CA, United States). The incorporation profiles were plotted and analyzed with the help of GraphPad^®^ (GraphPad Software^®^, Inc., CA, United States) or OriginPro 8.1^®^ (OriginLab Corporation^®^, MA, United States) software. All centrifugation steps were 30 s at 10,000 x *g* at room temperature. In all experiments, a control was performed where the traced solution was added, and immediately afterward the transport was stopped by the addition of ice-cold PBS (Time zero transport) and the values in CPMs of this sample were discounted from the others.

### Metabolic labelling of parasites

Within the three asexual stages, this work has been focused on the schizonts stage because previous studies related a higher incorporation rate of [^3^H] isoprenoid moieties at this stage (D'Alexandri et al., 2006; Kimura et al., 2011; Moura et al., 2001). For this, synchronous cultures of *P. falciparum* at the ring stage in 25 cm^2^ flasks were labelled with either 0.75 μCi/ml of each [^3^H] FOH, [^3^H] GGOH, or [^3^H] POH or 0.37 μCi/ml [^3^H] dolichol 95. [^3^H] FOH, [^3^H] GGOH, and [^3^H] POH were used directly from the ethanolic commercial solution. The necessary amounts of [^3^H] dolichol 95 dissolved in chloroform/methanol was dried and resuspended in ethanol. After 12–16 h, parasites at trophozoite/schizonts stages were obtained by saponin lysis (Christophers and Fulton, 1939). For this, cultures pellets were lysed with 30 ml of 0.03% saponin in PBS at 4°C. Parasites were then centrifuged at 1500 x *g* for 5 min at 4°C and subsequently washed in PBS. The number of cells was counted by microscopy and the material was immediately analyzed or stored in liquid nitrogen for later use. For all radiolabelling experiments, each sample was adjusted to contain 10^9^ cells.

### Analysis of parasitic phosphorylated isoprenoid alcohols

Radiolabelled parasites were extracted with 0.5 ml of n-butanol saturated in water. The sample was centrifuged for 20 min at 12,000 x *g*, and the organic phase was vacuum-dried and resuspended in 10 µL of n-butanol saturated in water. The samples and standards of those substances of interest were applied on a silica gel 60 TLC plates (Mallinckrodt Baker, Griesheim, Germany) and developed with isopropyl alcohol/ammonia (32%)/H_2_O (6:3:1 v/v/v) (Ischebeck et al., 2006). Finally, TLC plates were treated with En^3^HanceTM (Perkin Elmer^®^) and autoradiographed on photographic films for several days at -70°C. The identification of metabolites was performed by comparison with the R*f* of the internal standards, which were visualized with iodine vapor.

### Analysis of parasitic isoprenoid alcohols

Radiolabelled parasites were lysed with 2 ml of ice-cold 0.2 M perchloric acid in methanol and lipids were extracted with 4 ml of petroleum benzine. The sample was centrifuged for 10 min at 600 x *g*, and the organic phase was dried under a nitrogen stream and resuspended in 20 µL of hexane. The samples and standards of those substances of interest were applied on an RP-18 TLC plate (Mallinckrodt Baker, Griesheim, Germany) and developed with acetone/water (19:1 v/v) (D'Alexandri et al., 2006). The identification of metabolites was performed by comparison with the R*f* of the internal standards, which were visualized with iodine vapor. Finally, TLC plates were treated with En3HanceTM (Perkin Elmer^®^) and autoradiographed on photographic films for several days at −70°C.

### Preparation of food extracts

The foods used to prepare the extracts were sunflower oil (Liza, Cargill Agricolas S.A.^®^) and arugula (*Eruca sativa*), and the protocol used for extraction was according to de Wolf et al. (de Wolf et al., 2017). The extracts were prepared using 10 ml of sunflower oil or 50 g of arugula. The arugula has been liquefied with 100 ml of water and filtered in filter paper. After this, 10 ml of sunflower oil or arugula was transferred to a separating funnel and extracted with 60 ml methanol and 30 ml chloroform. The lower lipid phase was evaporated, and the residues were dissolved in 25 ml ethanol. These extracts were hydrolyzed with 25 ml of 5 M potassium hydroxide at 56°C for 1 h. After cooling and neutralization with 25 ml 5 M hydrochloric acid, the solution was extracted with 120 ml n-hexane, 30 ml water, and 30 ml ethanol. The upper organic phase was evaporated and dissolved in ethanol for analysis. For the drug-rescue assay using fosmidomycin, a fixed dose was used: 0.1 mg/ml for sunflower oil and arugula extracts.

### TLC chromatographic system for the separation of food isoprenoid alcohols

TLC RP-8 F254 (10 × 10 cm, Merck) plates were developed with a mixture of methanol:acetonitrile 1:1 (v:v). Alternatively, TLC RP-18 F245 (20 × 20 cm, Merck) plates were used, which were developed with an acetone:water 19:1 (v,v) mixture. In both cases, the identification of metabolites was performed by comparison with the *Rf* of the internal standards, which were visualized by iodine vapor and UV light.

### Drug-rescue assays in malaria parasites

The dose-response curve and the concentration of drug/metabolite required to cause a 50% reduction in parasite growth (IC_50_ value) were estimated in most cases by DNA staining as described elsewhere (Smilkstein et al., 2004). In some experiments the parasitemia was assessed by flow cytometry after 48 h, using SYTO11 as previously described (Urbán et al., 2011). Briefly, assays started at the ring stage, with 3% hematocrit, and 1% parasitemia after sorbitol synchronization at 37°C. A final amount of 200 μL of this *Plasmodium* culture, treated or not with antimalarials was plated in 96-well plates. The initial drug concentrations used were 20 µM for fosmidomycin and 1.6 µM for clindamycin in SYBR Green I^®^ DNA staining experiments and 100 µM for fosmidomycin in SYTO™ 11 staining experiments. Serial dilutions of the antimalarials were prepared in the 96-well microplates in RPMI complete medium supplemented or not with isoprenoid alcohols, solanesol, dolichols, or food extracts. After the corresponding incubation time, parasite growth was monitored by: 1) incubating 100 µM of the culture with 100 µM of an SYBR Green I^®^ DNA staining solution at 1:20,000 final dilution in lysis buffer (20 mM Tris, pH 7.5; 5 mM EDTA; 0.008% saponin (v/v); 0.08% Triton X-100 (v/v), the fluorescence was measured in a POLARstar Omega fluorometer^®^ (BMG Labtech^®^, Ortenberg, Germany) with the excitation and emission bands centered at wavelengths of 485 and 520 nm respectively; or 2) diluting each sample at 1:200 in an SYTO™ 11 (0.5 mM in DMSO) staining solution at 1:5,000 in PBS (final concentration of 0.5 μM). Samples were analyzed using BD LSRFortessa™ Cell Analyzer, set up with the standard configuration. Sample excitation was performed at 488 nm, air-cooled argon-ion laser at a power of 15 mW, using forward and side scatters to gate the erythrocyte population. SYTO 11 fluorescence (530 nm, FITC filter) was evaluated on a logarithmic scale. The single-cell population was selected on a forward–side scattergram, and the green fluorescence from the population was analyzed. Parasitemia was expressed in percentage.

Finally, the values obtained were subtracted from those values obtained for infected erythrocytes treat with 100 nM chloroquine or uninfected erythrocytes suspended at the same hematocrit. Solvent controls and untreated controls were always included and results were analyzed by GraphPad Prism^®^ software. All experiments were performed at least three times with four technical triplicates for each one.

### Gel electrophoresis

SDS-PAGE was performed in 12% gels as described elsewhere (Laemmli, 1970; Moura et al., 2001). Briefly, parasites were solubilized in SDS sample buffer, boiled for 5 min at 95°C, and applied to each well for analysis. All gels were treated with Amplify (Amersham), dried, and exposed to radiographic films with intensifying screen sets at −70°C.

### Proteomic studies

Two bands corresponding to the protein groups incorporating [^3^H] GGOH (band 1: 25-30 kDa; band 2: approximately 20–25 kDa) were excised from the acrylamide gel and cut into pieces of, approximately 1 mm^3^. Then, bands were washed with 40% acetonitrile in 50 mM ammonium bicarbonate. Each band was reduced with 10 mM DTT in 50 mM ammonium bicarbonate and incubated for 45 min at 56°C, alkylated with 55 mM iodoacetamide in 50 mM ammonium bicarbonate for 30 min at room temperature. Protein was digested into peptides by trypsin (Promega) at 1:50 (w/w) at 37°C for 16 h. The reaction was stopped by the addition of trifluoroacetic acid at a 1% final concentration. Then, 40% acetonitrile and 0.1% trifluoroacetic acid were added to the gel to extract peptides. The purified peptides were eluted on a C18 resin (Thermo Scientific) (Gobom et al., 1999; Larsen et al., 2002) dried under a vacuum, and stored at −20°C for further analysis. The samples were analyzed by the Biomass—Mass Spectrometry and Proteome Discovery Facility at *Centro de Facilidades à Pesquisa* (CEFAP) of the Institute of Biomedical Sciences of the University of São Paulo. The samples were analyzed by an EASY-nano LC system (Proxeon Biosystems) coupled with an LTQ-Orbitrap Velos mass spectrometer (Thermo Scientific). Peptides extracted from each fraction were loaded into an 18 cm silica column (100 μm internal diameter) with a 3 μm ReproSil-Pur C18-AQ reversed-phase capillary column (Dr. Maisch GmbH, Germany) and eluted using a gradient of 2–30% phase B (0.1% formic acid, 95% acetonitrile) for 80 min, reaching 95% phase B over 5 min and holding at 95%B for 20 min (one total 25 min at 300 nL/min). Mass spectra were acquired in positive ion mode by applying an automatic scan dependent on the acquired tandem mass spectrum (MS/MS) data. Each MS scan on the orbitrap (m/z mass range of 350–1500 and resolution 60,000) was followed by an MS/MS acquisition of the twenty most intense ions. Fragmentation was performed by collision-induced dissociation and sequenced ions were dynamically excluded for 60 s. The raw data from MS/MS analysis were visualized by the Xcalibur v.2.1 software (Thermo Scientific) and data processing was performed using Maxquant version 1.5.3.8 for protein identification and quantification. Raw files were generated and submitted to search using the Andromeda algorithm in the *P. falciparum* 3D7 clone database downloaded from UNIPROT (https://www.uniprot.org/, accessed April 2021). The identified proteins were filtered to contain a single peptide; at least two identified peptides had a molecular weight compatible with the gel bands. Finally, proteins containing prenylation domains were identified on the iPreny-PseAAC website (http://app.aporc.org/iPreny-PseAAC/index.html. Last accessed in 2020) and their weight and the isoelectric point were predicted in the UNIPROT database website. The mass spectrometry proteomics data is available for public access in the ProteomeXchange Consortium *via* the PRIDE partner repository with the dataset identifier PXD036092.

### 2D gel electrophoresis

Parasite pellets were suspended in 500 µL lysis buffer (2% SDS, 60 mM DTT in Tris/HCl pH 9) and heated at 95°C for 5 min. Then, the extract was centrifuged at 12,000 x *g* for 20 min, and proteins contained in the supernatant were precipitated with the 2-D Clean-Up Kit (Cytiva). Then, the protein was solubilized in 2D buffer (Bio-Rad) containing 0.2% (v/v) ampholytes pH 5/8 (Bio-Lyte 5/8 Ampholyte, Bio-Rad, #1631193). This material was used to rehydrate overnight a 7 cm IPG, pH = 5-8 L strip (Biorad). First-dimensional isoelectric focusing was performed on a Biorad Protean^®^ IEF Cell (Biorad). Isoelectric focusing was always allowed to proceed to a total of 15.000 V-h, which that completed within 3–4 h. IPG strips were equilibrated for 10 min each in SDS equilibration buffer (50 mM Tris-HCl, pH 8.8, 6 M urea, 30% (v/v) glycerol, 4% (w/v) SDS), 2% DTT). Finally, the strip was placed in an SDS electrophoresis running buffer (0.25 M Tris-HCl, pH 8.3, 0.1% SDS, 192 mM glycine) for 10 min as a final equilibration step. Second-dimensional separation was performed by placing the IPG strips on top of the 12% SDS PAGE gel. Separation was performed at 90 V as previously described. Gels were fixed in 45% (v/v) methanol, and 5% (v/v) acetic acid overnight, and proteins were observed by silver staining. Finally, gels were treated with Amplify (Amersham), dried, and exposed to radiographic films with intensifying screen sets at -70°C. This protocol was adapted from the methodologies of O’Farrell (O'Farrell, 1975) and Smit et al. (Smit et al., 2010).

### Sulfonium salt cleavage

Sulfonium salt cleavage of parasitic proteins was performed following a protocol adapted to what was described by Cenedella et al., (Cenedella, 1998). First, proteins of radiolabelled parasites were subjected to SDS-page electrophoresis as previously described. Radiolabelled proteins from gel bands were extracted for 2 h with 1.4 ml of 8 M urea at 100°C in a screw-capped test tube. The urea extract was allowed to cool on ice. Then, 42 µL of pure formic acid and 500 µL methyl iodide were added to the extract (1.4 ml) in a crew-capped test tube. The tube was flushed with nitrogen and incubated in the dark for 24 h at 37°C with rotatory mixing. The methyl iodide was removed under vacuum and the pH of the aqueous phase was adjusted to 11 with 6 N NaOH. Then, the extract was incubated for 24 h in the dark at room temperature. Cleaved isoprenoids were extracted with 2 ml of methanol and 4 ml of chloroform (4 ml). The chloroformic phase was washed with 4 ml of water till removing urea. Finally, the chloroformic phase was evaporated, dissolved in 15 µL of n-hexane, and applied on RP-8 F254 TLC plates (10 × 10 cm, Merck) which were developed with a 1:1 methanol: acetonitrile (v/v). The identification of metabolites was carried out by comparison with the R*f* of the internal standards, which were visualized through iodine vapor and UV light. Finally, TLC plates were treated with En^3^HanceTM (Perkin Elmer^®^) and autoradiographed on photographic films for several days at -70°C.

### NMR

The POH standard purchased from ARC^®^ (cod. 0286-1g) was dissolved in CDCl_3_ with tetramethylsilane (TMS, 0.05% (v/v)) an internal standard (TMS = 0.00 ppm). The ^1^H NMR spectra were recorded at a frequency of 500 MHz on a spectrometer DRX 500 Bruker. Scans were performed with pulses widths (PW) of 8.0 ls and relaxation delay (RD) of 6.0s. Data were processed using Mestre-C (version 4.8.6.0, MestreLab), and the FIDs were Fourier transformed with line broadening (LB) = 1.0 Hz. Spectra were referenced to TMS at 0.00 ppm. Spectral intensities (peaks) were integrated into regions of equal width (0.02 ppm) in the range of d 1.40–12.40. Integrated areas were normalized to the equal total area. The region d 7.20–7.30, containing the chloroform peak, was excluded from the analysis. Data were analyzed by considering the previous work of Arigoni et al. (Arigoni et al., 1999), and Schröder et al., (Schröder et al., 2014). (Supplementary Figures S1, S2).

### RP-HPLC

The following isocratic RP-HPLC system was based on the methodology employed by Frolik et al. for the study of retinoids (Frolik et al., 1978). However, the system proved to be also efficient to separate different polyprenols such as POH and GGOH. The system was performed at 25°C using acetonitrile: 1% ammonium acetate (4:1) at a flow rate of 2 ml/min. It was employed a Hichrom^®^ Nucleosil^®^ 100 C18 column (250 mm × 4.6 mm) (Leicestershire, UK), a diode array detector (DAD) 170, and a fraction collector FC203B purchased from Gilson^®^ (Villiers-le Bel, France). The software used for data processing was the Trilution™ LC 3.0 System Software^®^. Polyprenol’s elution was monitored at 220 and 350 nm and fractions were collected every 0.5 min. RP-HPLC fractions were dried at 50°C and then suspended in 0.5 ml of liquid scintillation mixture (PerkinElmer Life Sciences^®^, MA, United States). The radioactivity of each fraction was monitored with a Beckman LS 5000 TD *β*-counter scintillation counter (Beckman^®^, CA, United States). Metabolic profiles were analyzed by OriginPro 8.1^®^ software (OriginLab Corporation^®^, MA, United States).

## Results

### Transport of isoprenoid alcohols

**FIGURE 2:**
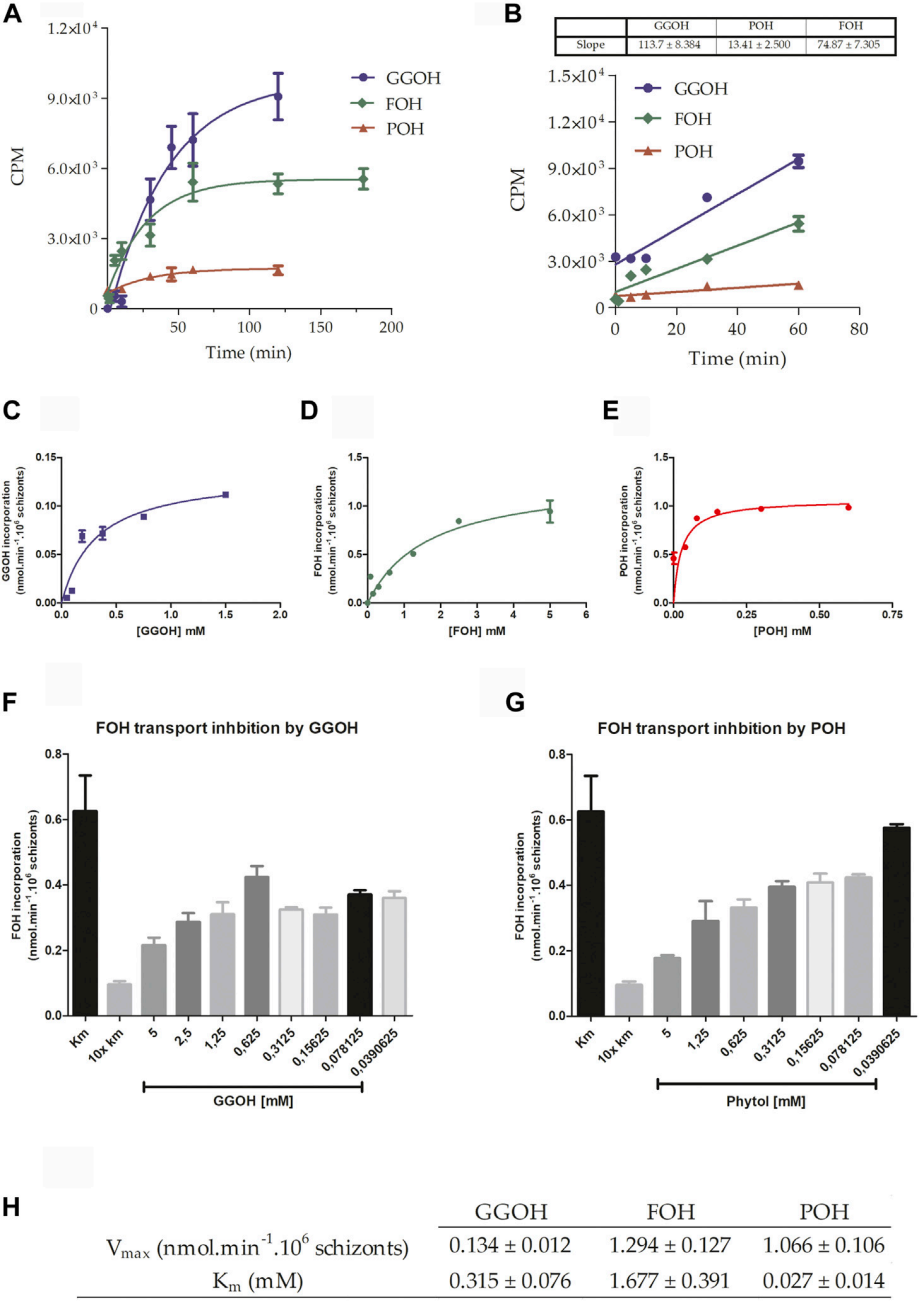
Characterization of isoprenoid alcohols’ transport. **(A)** The incorporation of 0.5 mM of GGOH or POH and 1 mM of FOH traced with the respective radiolabelled molecules was measured for 120 min. In **(B)**, the slope of the transport process up to 60 min was estimated using a linear regression equation. With this data, it was possible to calculate de V_0_ (initial velocity), fundamental to verify the effect of the concentration on the transport of **(C)** GGOH, **(D)** FOH, and **(E)** POH. After that, the transport of FOH (at the concentration corresponding to K_m_: 1.7 mM) was measured in the presence of different concentrations of **(F)** GGOH or **(G)** POH, presenting a concentration-dependent inhibition profile. An inhibition control was used as an excess of non-radioactive FOH. In the end, the kinetic parameters were summarized in **(H)**.

Initially, the parasite’s ability to incorporate the isoprenoid alcohols was verified. As can be seen in Figure 2A, parasites are capable of uptaking FOH, GGOH, and POH from the extracellular medium, presenting a saturable profile of temporal incorporation that adjusts to an exponential decay function, which indicates the intermediation of a carrier, in other words, this is not a passive diffusion through all the membranes. Near-linear incorporation was obtained during the first 60 min for the isoprenoid alcohols (Figure 2B). Based on this data, it was assumed that V_0_ (initial transport velocity) could be calculated by measuring each isoprenoid alcohol incorporation for 10 min, and it was possible to calculate the velocities of the transport. To calculate the kinetic parameters for all isoprenoid alcohol’s transport, V_0_ was measured as a function of each substrate concentration. Data related to the transport of GGOH (Figure 2C), FOH (Figure 2D), and POH (Figure 2E) were adjusted to a classical Michaelis–Menten function. GGOH presented the average V_max_ of 0.134 nmol/min, approximately 10x and 8x lower than an average V_max_ of FOH (V_max_ = 1.07 nmol/min) and POH (V_max_ = 1.29 nmol/min), respectively. Regarding the values of K_m_, POH has the lowest mean value (0.027 mM), which accounts for about 12x and 62x less than the mean K_m_ of FOH (K_m_ = 1,677 mM) and GGOH (K_m_ = 0.315 mM).

Figures 2F,G show that FOH transport could be inhibited by increasing concentrations of the other two isoprenoid alcohols, respectively GGOH and POH, suggesting that the same transport system could transport these molecules. With the V_0_ calculated previously, the kinetic parameters V_max_ and K_m_ were estimated, and it can be indicated that the transport system has its highest velocity when FOH is the substrate but has a greater affinity to POH (Figure 2H).

### The metabolization of isoprenoid alcohols

**FIGURE 3:**
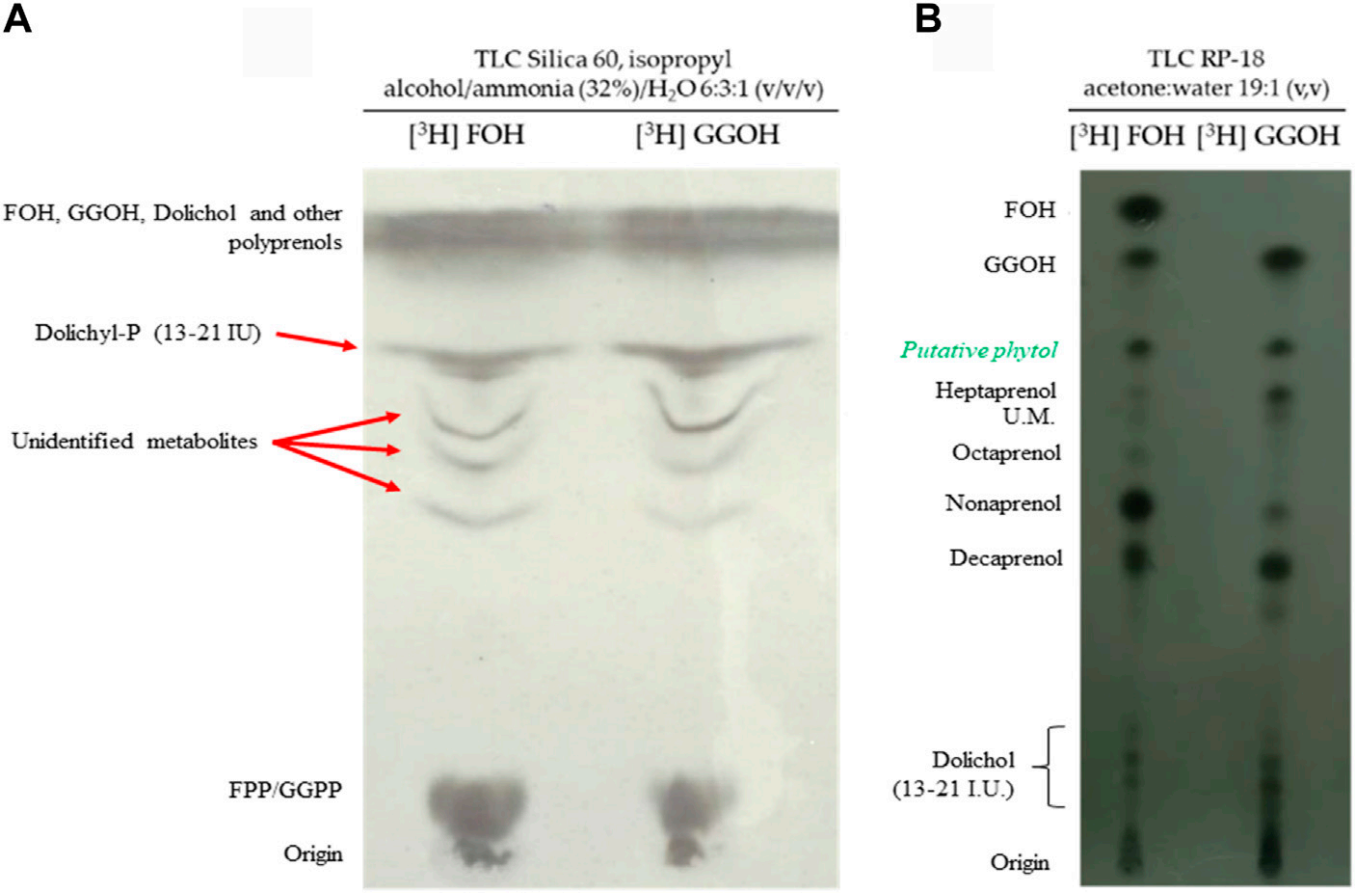
The metabolization of isoprenoid alcohols. **(A)** Autoradiography of a TLC analysis (TLC plates silica gel 60 developed with isopropyl alcohol/ammonia (32%)/H_2_O (6:3:1 v/v/v) of the lipids released from parasites incubated with [^3^H] FOH or [^3^H] GGOH. **(B)** Autoradiography of a TLC analysis (RP-18 plates developed with acetone/water 19:1; v/v) of the lipids released from parasites incubated with [^3^H] FOH or [^3^H] GGOH.

The natural substrates of polyprenyl transferases and polyprenyl synthetases are isoprenoid pyrophosphates (FPP, GGPP or longer polyprenyl pyrophosphates) (Crick et al., 1997; Verdaguer et al., 2019). Interestingly, their alcohol counterparts (FOH and GGOH) can rescue parasites from fosmidomycin, suggesting that the parasite possesses a mechanism to phosphorylate them (Yeh and DeRisi, 2011; Guggisberg et al., 2016; Kennedy et al., 2019). Infected erythrocytes were incubated with [^3^H] FOH, [^3^H] GGOH, and the parasites were isolated by using saponin lysis protocol. The samples were so subjected to extraction with n-butanol saturated in water and analyzed through a chromatographic system containing ammonia. Under these conditions, ammonium salts of phosphates and pyrophosphates are formed providing the separation of unphosphorylated substances from dolichyl-P and polyprenyl pyrophosphates such as FPP or GGPP (Ischebeck et al., 2006). As can be seen in Figure 3A, it was possible to detect phosphorylated forms derived from FOH/GGOH. This finding confirmed previous results (Yeh and DeRisi, 2011; Guggisberg et al., 2016; Kennedy et al., 2019) and indicates that *P. falciparum* possesses an enzymatic system to phosphorylate FOH/GGOH into their pyrophosphate forms. This phosphorylation process was also suggested in *P. falciparum* by employing POH as a substrate (Sussmann et al., 2022). Besides it, other radiolabelled apolar substances were detected. Some of these substances were coincident with components of the dolichyl-P 13–21 mixture standard (Figure 3A).

Since FOH and GGOH can be phosphorylated and recover the effect of fosmidomycin, it was verified if the parasite is able to use these molecules to synthesize longer isoprenoids. For this, was used a chromatographic system capable of separating several isoprenoid alcohols with a sufficient resolution. Unfortunately, this method does not directly detect the phosphorylated isoprenoids and so the radiolabelled parasites had to be subjected to an extraction under acidic conditions to promote the dephosphorylation before the analysis (Popjak et al., 1961). Therefore, each band detected in Figure 3B corresponds to a mixture of isoprenoid alcohol and its phosphorylated counterpart. As can be seen in Figure 3B the parasite converted FOH and GGOH into different longer isoprenoids. Those molecules were compatible with polyprenol of 7–10 isoprenic units and dolichols. Interestingly, the conversion of FOH and GGOH to a molecule compatible with POH was also observed. It was also observed that GGOH incorporates better into nonaprenol rather than octaprenol and FOH the opposite. Coincident with this, it was previously reported that [^3^H] GGPP is better incorporated into ubiquinone-9, while [^3^H] FPP appeared to be better incorporated into ubiquinone-8 (Verdaguer et al., 2021). These two observations suggest that the distinct polyprenyl synthases possess different levels of specificity and affinity according to the isoprenic chain of the substrate. Furthermore, FOH was converted into GGOH/GGPP (Figures 3A,B).

### Utilization of isoprenoid alcohols for post-translational modification of proteins

**FIGURE 4:**
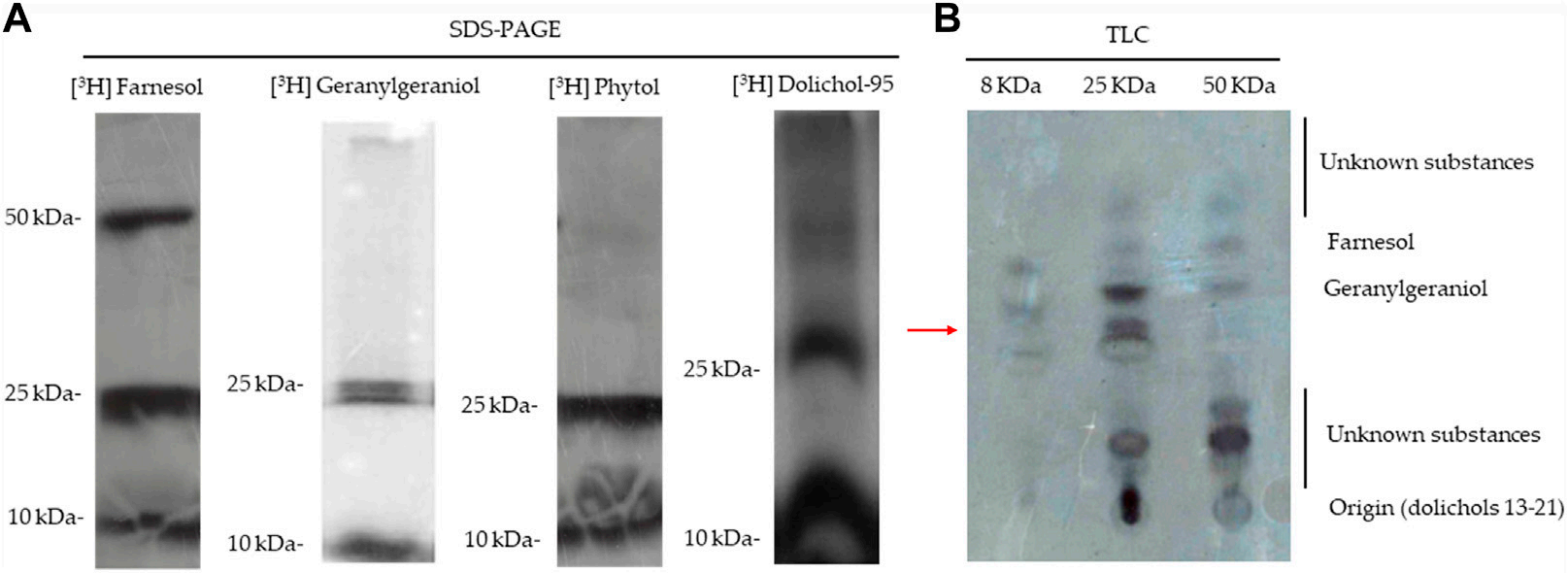
SDS-PAGE and iodomethane treatment of prenylated proteins. **(A)** SDS-PAGE analysis of [^3^H] GGOH, [^3^H] FOH, dolichol-95, and POH-labeled proteins of *P. falciparum*. **(B)** Substances released from the cleavage with methyl iodide of the three protein clusters incorporate [^3^H] FOH; (autoradiography of a TLC analysis). The chromatographic retention of several isoprenoid alcohol standards is indicated in the figure. Red arrow: *Rf* of POH.

The disruption of the digestive vacuole function has been associated with the loss of parasitic homeostasis due to fosmidomycin treatment (Yeh and DeRisi, 2011). The cause of this seems to be the loss of protein prenylation, which abrogates the function of vesicular trafficking proteins, preventing their membrane attachment and the consequent feeding/nutrition of malaria parasites (Kennedy et al., 2019). Considering this, we evaluated the identity of isoprenoids bound to proteins. Parasites were radiolabelled with [^3^H] FOH, [^3^H] GGOH, [^3^H] POH, or [^3^H] dolichol 95, and all radiolabelled isoprenoid alcohols were found in an ∼8 kDa protein cluster and at least two bands contained in a ∼25 kDa protein cluster. However, only FOH was incorporated into another cluster of ∼50 kDa (Figure 4A). This pattern agrees with the results previously described by D'Alexandri et al. by employing [^3^H] GGPP and [^3^H] FPP (D'Alexandri et al., 2006). It was further studied that the isoprenoids bound to [^3^H] FOH radiolabelled protein clusters by cleaving the bounded lipids with iodomethane (Figure 4B). Results showed that ∼8 kDa and ∼25 kDa clusters contain proteins modified by FOH and GGOH while the ∼50 kDa protein cluster almost does not contain geranylgeranylated proteins. Very apolar substances such as dolichols were only detected in the ∼25 kDa cluster (at the origin of the TLC plate) and unidentified substances were found in all the clusters analyzed (Figure 4B). These unidentified substances did not migrate with polyprenols of 8–10 isoprenic units. However, one of the unidentified substances was compatible with POH (the more apolar unidentified substance bound to the ∼25 kDa cluster; see Figure 4).

**FIGURE 5:**
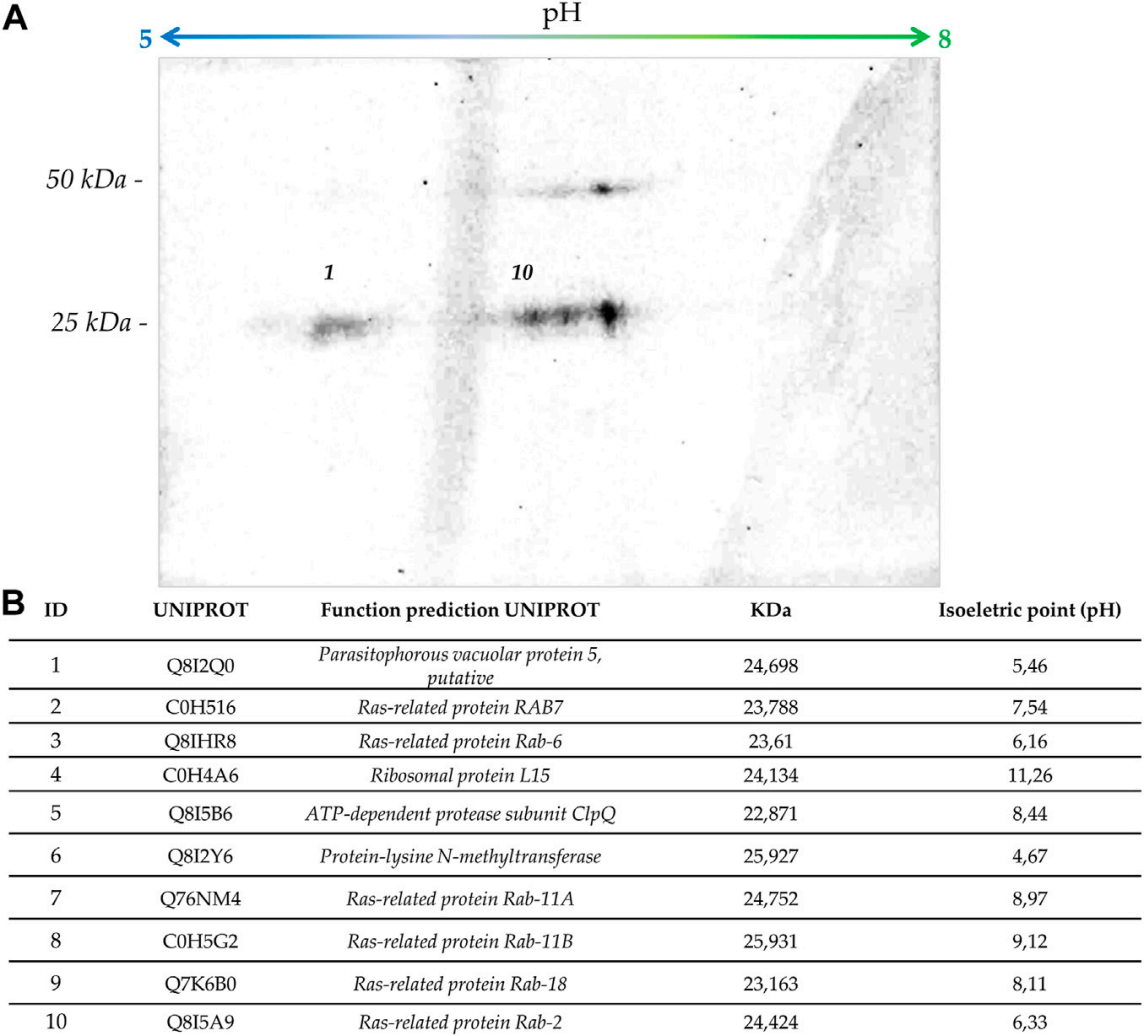
Proteomic analysis. **(A)** The figure shows an auto-radiography of 2D gels with [^3^H] FOH-labelled proteins of *P. falciparum*. **(B)** The table indicates those ∼25 kDa prenylated proteins identified by mass spectrometry together with their molecular weight and isoelectric point prediction.

The identity of the ∼25 kDa prenylated proteins was further investigated through mass spectrometry-based proteomic approaches. Bands from SDS-PAGE containing parasitic proteins were excised from 25–30 kDa (band 1) and 20-25 kDa (band 2), in-gel digested, and analyzed by mass spectrometry. A total of 234 proteins were identified, 225 in band 1 and 202 in band 2. Most of the proteins were present in both bands, probably due to an insufficient electrophoretic separation of proteins. The analysis of the sequences on the iPreny-PseAAC website (http://app.bioinfoamss.org/iPreny-PseAAC/index.html) identified 10 proteins with a prenylation domain in band 1 and just one protein with a prenylation domain in band 2. Most of the identified proteins belong to the group of Ras-related proteins (also known as Rap proteins) (see table in Figure 5). Other proteins were identified, such as a protein from the parasitophorous vacuole (putative), the ClpQ subunit of an Adenine Triphosphate (ATP)-dependent protease, the ribosomal protein L15, and an N-methyltransferase. By performing 2D gel electrophoresis of [^3^H] FOH labelled proteins, it was possible to identify four spots, two of them could be identified as compatible with Protein 5 from the parasitophorous vacuole (putative; Uniprot: Q8I2Q0; PlasmoDB: PF3D7_0925900) and Ras-related protein Rab-2 (Uniprot: Q8I5A9; PlasmoDB: PF3D7_1231100) (Figure 5). Therefore, parasites potentially modify the same proteins with different types of isoprenoids, including dolichols and GGOH. Therefore, the results presented here suggest that: 1) the plasmodial farnesyltransferase uses other unidentified substrates, in addition to its natural substrate, FPP to modify proteins; 2) the plasmodial geranylgeranyltransferase uses POH, dolichols in addition to its natural substrate, GGPP to modify proteins. These two membrane-bound enzymes are thought to catalyze a post‐translational modification of proteins named isoprenylation. In the *P. falciparum* genome database, there are annotations for a putative multimeric protein farnesyltransferase (PF3D7_1242600; which non-specifically recognizes CAAX motifs in which X is methionine, glutamine, serine, threonine, or cysteine) and a putative multimeric protein geranylgeranyltransferase type II (PF3D7_0602500; an enzyme which recognizes CAA-Leucine motifs). As previously commented, only 20-carbon isoprenoid alcohols (GGOH and POH) possess the ability to completely rescue fosmidomycin at micromolar concentrations in the first intraerythrocytic cycle, which indicates the special importance of proteins modified by 20-carbon isoprenoids to maintain homeostasis in parasites exposed to fosmidomycin other drugs that affect the apicoplast integrity.

### Phytol recovers fosmidomycin and clindamycin antiplasmodial effect

**FIGURE 6:**
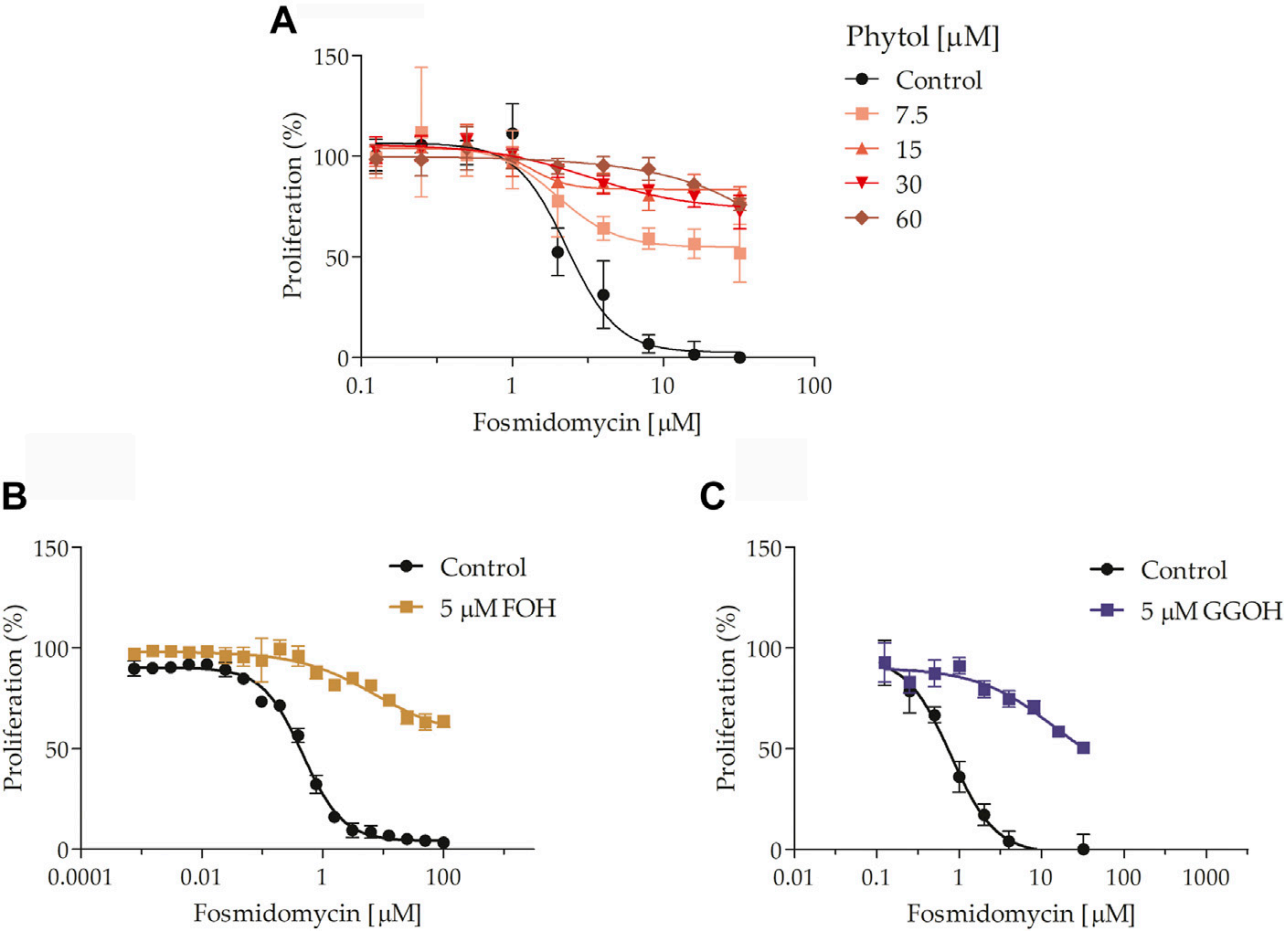
Fosmidomycin-rescue assays using isoprenoid alcohols. It was studied the fosmidomycin dose-response effect at 48 h in the presence or absence of 7.5, 15, 30, or 60 μM POH **(A)**, 5 μM FOH **(B)**, and 5 μM GGOH **(C)**. Fosmidomycin IC_50_ was calculated in **(A)** 1.903 ± 0.519 µM, **(B)** 1.220 ± 0.604 µM and **(C)** 1.250 ± 0.438 µM. The value represents the mean ± SD from three independent assays. Statistical analysis was performed with One Way ANOVA, followed by the Tukey posttest (***p < 0 .005 in relation to control).

Thereafter, it was explored which isoprenoid alcohols could attenuate fosmidomycin’s toxic effects in *P. falciparum*. For this purpose, fosmidomycin IC_50_ value at 48 h was calculated in the presence or not of 0.9 mg/ml dolichol 13-21 (maximum non-toxic concentration for the parasite; see Supplementary Data; Supplementary Figure S3), 5 µM FOH, 5 µM GGOH, 7.5–60 µM POH (non-toxic concentrations for the parasite; see Supplementary Data; Supplementary Figure S3), and 5 µM solanesol. After several attempts, it was not possible to rescue parasites of fosmidomycin toxicity by employing the mixture of dolichols 13-21 (see Supplementary Data; Supplementary Figure S4). On the other hand, POH (Figure 6A) rescued the fosmidomycin effect in a dose-dependent manner at concentrations up to >4 µM of the antimalarial. FOH and GGOH also limited the fosmidomycin effect as expected (Figures 6B,C respectively). As far as we know, this is the first time that is observed the rescue of parasites from fosmidomycin toxicity by employing POH. Importantly, POH toxic effects for the parasite were only observed at concentrations >60 μM, and its IC_50_ value was estimated to be around 160 μM (see Supplementary Data; Supplementary Figure S3).

**FIGURE 7:**
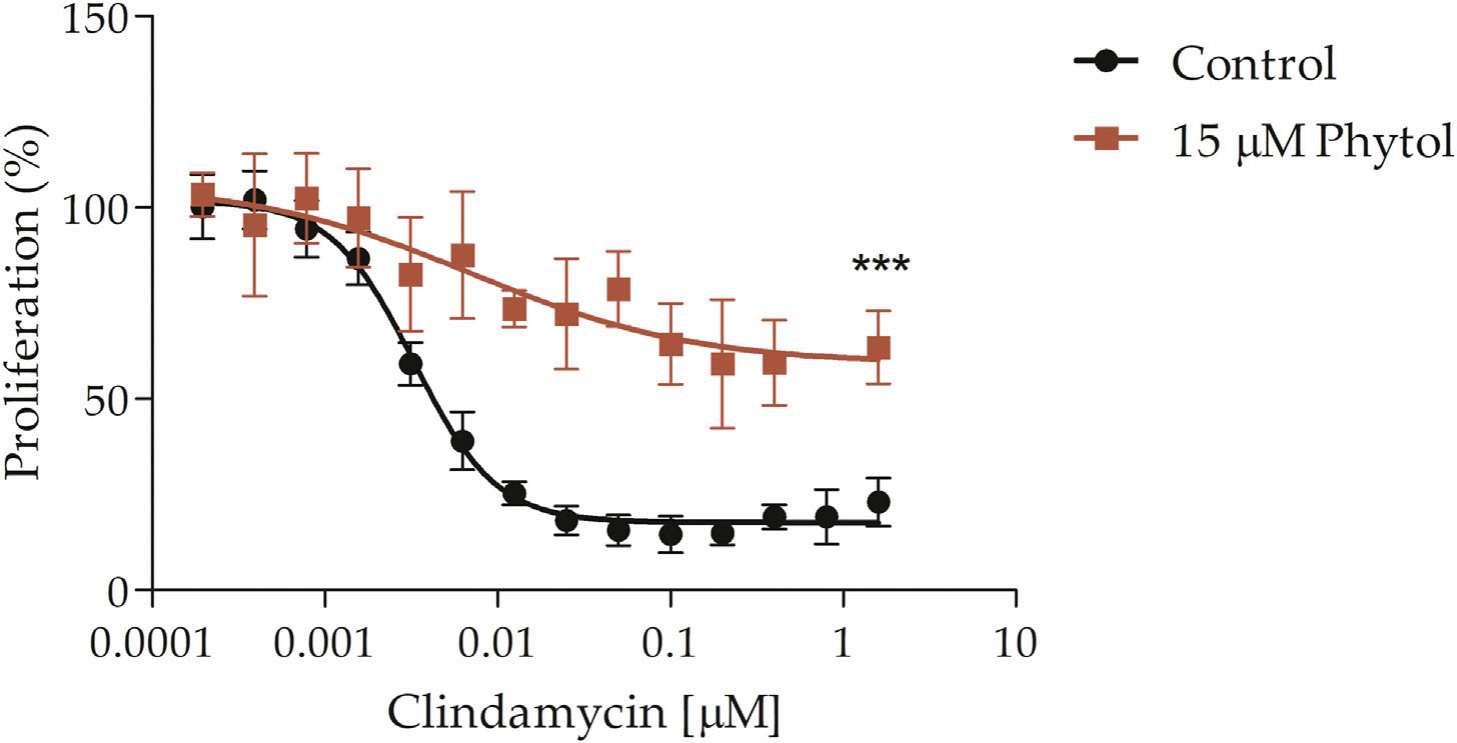
Clindamycin-rescue assay. Clindamycin dose-response effect at 72 h in the presence or absence of 15 μM POH. Clindamycin IC_50_ = 4.8 ± 1.1 nM. The value represents the mean ± SD from two independent assays. Statistical analysis was performed with One Way ANOVA, followed by the Tukey posttest (*** *p* < 0 .005 compared to the control).

It was further explored if POH may also rescue the effect of ribosomal inhibitors in *P. falciparum*, as it was previously reported for IPP and GGOH (Hjertman et al., 1997; Dahl and Rosenthal, 2007; Yeh and DeRisi, 2011; Kennedy et al., 2019). For this purpose, the clindamycin antimalarial effect was evaluated in the presence or absence of 15 μM of POH (Figure 7). POH significantly reduced the clindamycin effect 2 to 3 times at the concentration of 1.6 μM. The assay was performed at 72 h since clindamycin produces a delayed dead effect in the parasite (Fichera and Roos, 1997; Dahl and Rosenthal, 2007; Goodman et al., 2007) Finally, it is important to comment that we observed these rescue effects by employing the POH standards purchased from American Radiolabelled Chemicals, Inc. (ARC) (as previously shown) and Merck KGaA^®^ (see the commercial details in the methodological section). However, we have observed that POH standards purchased from other companies do not rescue the toxic effects of fosmidomycin by employing the cytometry method and microscopy of blood smears. Commercial POH can present important differences in its cis/trans ratio composition as well as several degradation products due to extractive procedures (Schröder et al., 2014). Considering this, we provide an HPLC and NMR analysis of the POH purchased from ARC, which was employed for all experiments here exposed. We also provide an HPLC analysis of the radiolabelled POH further employed for experiments (see Supplementary Data, Supplementary Figures S1, S2, respectively).

### Food extracts recover the fosmidomycin effect

**FIGURE 8:**
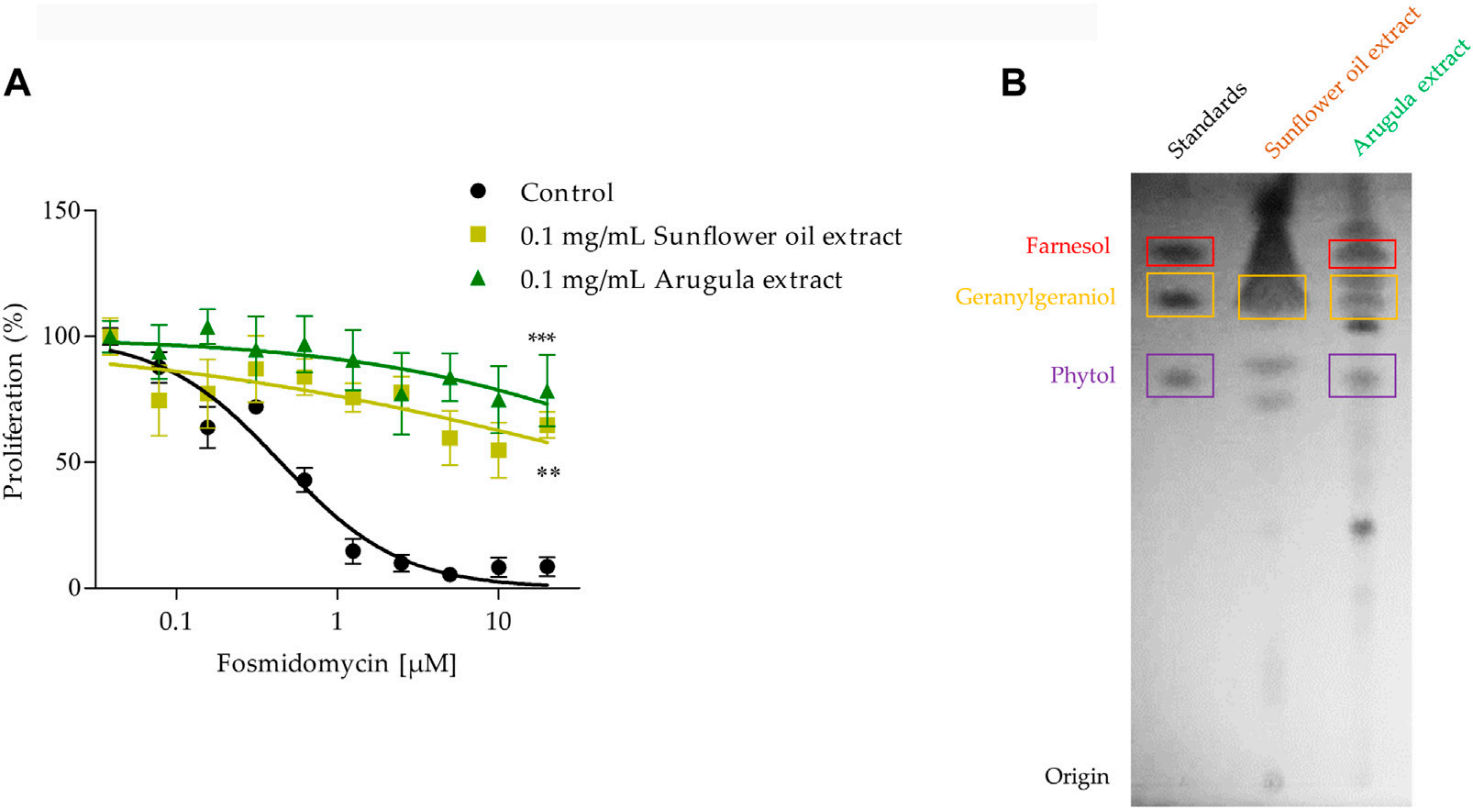
Fosmidomycin-rescue assays using sunflower oil and arugula extracts. **(A)** It was studied the fosmidomycin (initial concentration 20 µM) dose-response effect at 48 h in the presence or absence of 0.1 mg/ml sunflower oil and arugula extracts. Fosmidomycin IC_50_ = 0.7µM ± 0.3. These experiments were carried out using the SYBR Green I^®^ DNA staining methodology. Values represent the mean ± SD from three independent assays. Statistical analysis was performed with One Way ANOVA, followed by the Tukey posttest (*** *p* < 0.005 compared to the control). In **(B)** the TLC chromatographic analysis of the different extracts is shown as well as the FOH, GGOH and POH standards.

Some foods that are part of the human diet are rich in isoprenoid alcohols, and as can be seen in our previous experiments, some of these molecules can interfere with the antimalarial activity of fosmidomycin. Based on this, sunflower oil and arugula were chosen to be used in this work. It has previously been described that a range of food extracts (oils, meals, fruits, and vegetables) contains geranylgeraniol and recover the effect of statins in human cells, including sunflower oil and vegetables (de Wolf et al., 2017; Jawad et al., 2022). The antimalarial effect of fosmidomycin was evaluated at 48 h in the presence or not of 0.1 mg/ml sunflower oil or arugula extract. Both extracts recovered the effect of fosmidomycin, which can be observed in Figure 8A. The average IC_50_ calculated value of fosmidomycin alone was approximately 0.7 µM. Since the recovery of the fosmidomycin effect occurred up to high concentrations, it was not possible to estimate the IC_50_ in the presence of the extracts, but both extracts significantly reduced the fosmidomycin effect by approx. 5.5 times at the concentration of 20 μM. The recovery experiment was also performed by flow cytometry, with similar results (reduction of approx. 3 times using sunflower oil extract and 5 times using 5 µM of GGOH as control), as can be seen in Supplementary Figure S5. We were also able to identify compatible compounds (with the same *Rf*) with GGOH in both extracts and POH in arugula extract (Figure 8B).

## Discussion

One of the most promising targets for malaria drug design is the isoprenoid biosynthesis by the MEP pathway located in the apicoplast. This pathway can be inhibited by two drugs, fosmidomycin or its analogues and ribosomal inhibitors (Ex. doxycycline, clindamycin, and azithromycin) (Dahl and Rosenthal, 2007; Jackson and Dowd, 2012). Fosmidomycin inhibits the DXR enzyme and the ribosomal inhibitors interfere with apicoplast biogenesis and because of this, act slowly and possess poor antimalarial activity if not combined with other antimalarial drugs (Dahl and Rosenthal, 2007). Similarly, several human fosmidomycin clinical trials have failed to cure malaria due to recrudescence phenomena even when combined with other antimalarials (Borrmann et al., 2006; Lanaspa et al., 2012; Fernandes et al., 2015). Therefore, it is necessary to investigate better the apicoplast-targeting antimalarials mechanism of action and its effects on the parasite.

The MEP pathway leads to the formation of isoprenic units, necessary for the formation of several metabolites such as ubiquinones, prenylated proteins, and dolichols (D'Alexandri et al., 2006; Verdaguer et al., 2021; Mathews et al., 2019; Moura et al., 2001). It is also known that *P. falciparum* produces dolichols of 11–12 and 15-19 isoprenic units which are involved in protein glycosylation as well as protein dolichylation (D'Alexandri et al., 2006; Zimbres et al., 2020). The toxic effect of both drugs by the parasite can be rescued by isoprenoid alcohols such as FOH and GGOH (Guggisberg et al., 2016; Kennedy et al., 2019; Mathews et al., 2019). These metabolites are efficiently incorporated by the parasite and subsequently phosphorylated and employed as substrates for protein prenylation, for the biosynthesis of dolichols, and other isoprenoid alcohols such as nonaprenol, required for ubiquinone biosynthesis. Isoprenoid alcohols phosphorylation and parasite utilization indicate the presence of, at least, one still unknown polyprenol kinase that would convert FOH and GGOH into their active pyrophosphorylated forms (Crick et al., 1997) and a novel salvage pathway utilizing FOH and GGOH for protein prenylation. This kinase reaction was previously suggested in parasitic extracts by employing POH as a substrate (Sussmann et al., 2022). Biochemical evidence for a metabolic pathway for 15-20 carbon isoprenoid alcohol phosphorylation had been found in several organisms including Archaebacteria (Ohnuma et al., 1996), animal tissues (Bentinger et al., 1998), and plants (Ischebeck et al., 2006; Fitzpatrick et al., 2011; Vom Dorp et al., 2015). However, only in plants were identified proteins responsible for this pathway (Valentin et al., 2006; Fitzpatrick et al., 2011; Vom Dorp et al., 2015). In all cases cited, there are two enzymes for the consecutive phosphorylation of 15/20 carbon polyprenols to polyprenyl-PP. In *Plasmodium* no enzyme responsible for this pathway was identified yet.

It is important to highlight that we could verify in this study that the effect of inhibition of plasmodial isoprenoid biosynthesis is reversed by the addition of food extracts. This effect was previously observed by de Wolf et al. in human cells and *in vivo* models (de Wolf et al., 2017). The authors concluded that the anti-cancer effects of pitavastatin were limited due to the dietary GGOH. This raises the concern that dietary isoprenoid alcohols could also interfere with the antimalarial activity of fosmidomycin and/or ribosomal antibiotics in clinical trials. Further studies are necessary to better investigate if dietary isoprenoid alcohols can limit the activity of certain antimalarial treatments.

The loss of protein prenylation is the primary cause of the death of parasites due to fosmidomycin or ribosomal inhibitors (Kennedy et al., 2019). This post-translational modification of proteins occurs by a thioether linkage of isoprenoid alcohol to cysteine residues of proteins (Epstein et al., 1991). In *P. falciparum*, most prenylated proteins are involved in vesicular trafficking (e.g. Ras and Rap proteins) and, thus, are essential for feeding malaria parasites (Chakrabarti et al., 1998). Here, we demonstrated that not only GGOH and FOH can be used for protein prenylation but also exogenous dolichols and POH, an acyclic diterpene produced through the hydrogenation of GGPP (Hansen, 1980). Some authors suggest the parasite uses POH for the biosynthesis of some vitamins in the parasite (Sussmann et al., 2011; Sussmann et al., 2022). In nature, POH can be obtained through two pathways. The first is the activity of a chlorophyll synthase, an NADH-dependent enzyme that reduces the pyrophosphate ester of GGOH moieties attached to chlorophyll (Chl) biosynthesis intermediates (Shalygo et al., 2009). The other pathway directly hydrogenates GGPP by the action of a geranylgeranyl reductase (GGR; EC:1.3.1.83), present in some archaea, algae, plants, cyanobacteria, and bacteria species (Sasaki et al., 2011; Meadows et al., 2018). Although it has already been indicated the existence in plants (Parmryd et al., 1999), no phytylation activity or POH biosynthesis in non-photosynthetic organisms have been described in the literature. Nevertheless, human plasma contains POH from dietary sources. Human excretion physiology and food content have been exhaustively studied since Refsum disease is caused by excessive accumulation of its derivative, phytanic acid, in the plasma due to autosomal recessive mutations (Steinberg et al., 1966; Baxter, 1968; Wanders et al., 2001).

In the parasite, Sussmann et al. observed the incorporation of exogenous [1-(n)-^3^H]-POH (Sussmann et al., 2011; Sussmann et al., 2022). However, the ability of the parasite to produce POH or even obtain it from the exogenous environment is not understood. Here, we studied the utilization of POH in malaria parasites. Through radiolabelling and drug-rescue assays, it was observed that exogenous POH can be used for protein prenylation, similarly to GGOH. In addition, dolichols showed to bind to the same protein clusters as POH and GGOH. Besides POH, we show that protein dolichylation occurs naturally in the parasite by studying [^3^H] FOH labelled compounds attached to proteins. For this, sulfonium-salt cleavage with methyl iodide released compounds chromatographically compatible with dolichols, FOH, GGOH, and POH. However, it has been shown that sulfonium salt cleavage gives rise to hydrophobic rearrangement products of polyprenols (Whyte et al., 1997). It is thus possible that some of the observed are artifacts and are currently under investigation in our laboratory. Until now, only dolichol-11 attachment to parasitic proteins was already demonstrated by mass spectrometry techniques by D'Alexandri et al., in 2006 (D'Alexandri et al., 2006). The authors found that dolichol of 11 isoprene units is bound to 21-28-kDa protein clusters from trophozoite and schizont stages. Furthermore, the analysis of the proteolytic digestion products from parasite proteins labelled with [^35^S]-cysteine and [^3^H]-FPP showed that the attachment of dolichols to protein is a post-translational event and probably occurs *via* a covalent bond to cysteine residues. This post-translational modification was observed only in malaria parasites and once in human colon carcinoma cells (D'Alexandri et al., 2006; Hjertman et al., 1997). Therefore, radiolabelling of proteins with dolichols, shown here, represents further evidence for this post-translational modification. All results presented suggest the presence of two different promiscuous protein prenyltransferase in the parasite: one enzyme which uses FPP among other unidentified substrates and another enzyme that attaches proteins to GGOH, POH, and dolichols among other substrates not identified here. In *P. falciparum,* these post‐translational modifications of proteins are thought to be catalyzed by two membrane-bound enzymes: a putative multimeric protein farnesyl transferase (PF3D7_1242600; which nonspecifically recognizes CAAX motifs) and a putative multimeric protein geranylgeranyl transferase type II (PF3D7_0602500; an enzyme which recognizes CAA-Leucine motifs) (Farnsworth et al., 1990; Chakrabarti et al., 1998).

As commented previously, FOH and GGOH can rescue parasites lacking isoprenoid biosynthesis. Considering this, several authors interpreted that these isoprenoid alcohols supply protein geranygeranylation and farnesylation (Guggisberg et al., 2016; Kennedy et al., 2019; Mathews et al., 2019). However, our results demonstrate that other [^3^H] FOH-derived substances such as dolichols can be detected in the ∼25 kDa cluster together with other unidentified substances. The identity of the ∼25 kDa prenylated proteins was further investigated through proteomic approaches. Results detected fewer proteins with prenylation domains than what the parasite should express (Suazo et al., 2016). Probably, we identified the most abundant prenylated proteins of late trophozoite/schizont stages. Of all these proteins, only a few were identified by 2D gel electrophoresis of [^3^H] FOH-labelled parasitic cell extracts. This indicates that the parasite probably naturally modifies the same proteins with different types of isoprenoids, including dolichols and GGOH. We tested the hypothesis that dolichols or even nonaprenol may rescue fosmidomycin toxicity. However, only FOH, GGOH, and POH but not dolichols nor solanesol attenuate the antiplasmodial efficacy of the fosmidomycin. This observation suggests that dolichols and nonaprenol do not restore protein prenylation and that only the disruption of those proteins attached to 20-carbon isoprenoids is the primary cause of death of parasites due to fosmidomycin. This result also raises the question about the biological relevance of protein dolichylation. It could be suggested that only proteins attached to 20-carbon isoprenoids are functional compared to dolichylated proteins. Another possibility is that the isoprenoid molecule attached to proteins makes them localize to different cellular compartments and exert different biological functions. In this case, only proteins attached to 20-carbon isoprenoids would arrive in the correct subcellular compartment and accomplish essential functions for maintaining parasitic homeostasis.

Besides protein prenylation, it remains poorly understood the essentiality of dolichols biosynthesis by the parasite. Considering the ability of parasites to incorporate and use exogenous dolichols it is suggested that *P. falciparum* may rely on plasma lipids or erythrocyte membranes which are rich in dolichols of 18–20 isoprene units (Zimbres et al., 2020). Otherwise, Zimbres et al. suggested the parasite could possess a divergent protein N-glycosylation pathway that does not require dolichyl-P but polyprenols or other lipids as sugar transporters (Zimbres et al., 2020). The authors showed that inducible knockdown parasites in those *loci* encoding the enzymes *cis*-prenyltransferase and polyprenol reductase strongly affected dolichol biosynthesis without losing cellular viability. Furthermore, these knockdown parasites presented no mislocalization of the merozoite surface protein 1, a well-studied protein that is modified by glycosylphosphatidylinositol (Zimbres et al., 2020). Taken together, the data presented here will help to understand the apicoplast-targeting antimalarials mechanism of action better. They would be a starting point for studying a set of novel post-translational modifications of proteins in the parasite.

## Data Availability

The data presented in the study are deposited in the ProteomeXchange Consortium *via* PRIDE partner repository, accession number PXD036092.
