# Supplementary material for: Isoprenoid alcohols utilization by malaria parasites
Source: Front Chem. 2022 Dec 1;10:1035548. doi: 10.3389/fchem.2022.1035548 (PMC9751614; doi:10.3389/fchem.2022.1035548)
Supplement: Supplementary file 1 [file DataSheet1.pdf]

Supplementary Material

S1. NMR. POH purchased from ARC® was subjected to Nuclear Magnetic Resonance (NMR) studies. The ^{1}H NMR spectrum of POH (figure S1 b) had signals attributable to what was described by Arigoni et al., [42] for the *trans* configuration of isoprene (see chemical formula of *cis* and *trans* isoprene in figure S1 a). These signals include peaks at: 4.14 ppm for H-1, 5.41 ppm for H-2, 1.99 ppm for H-4 and 1.67 ppm for the methyl group. The *cis* configuration showed distinct signals for H-1 (δH 4.12 ppm), H-4 (δH 2.06 ppm) and for CH3 (δH 1.73 ppm) (see figure S1 b). Using the same parameters, the amount of *trans*-POH was foun to be ~97% (1,67 ppm). *Trans*-POH is the compound observed in nature and extractive procedures convert it into several compounds, such as *cis*-POH, isophytol, and dihydrophytol which may constitute the principal contaminants [43].

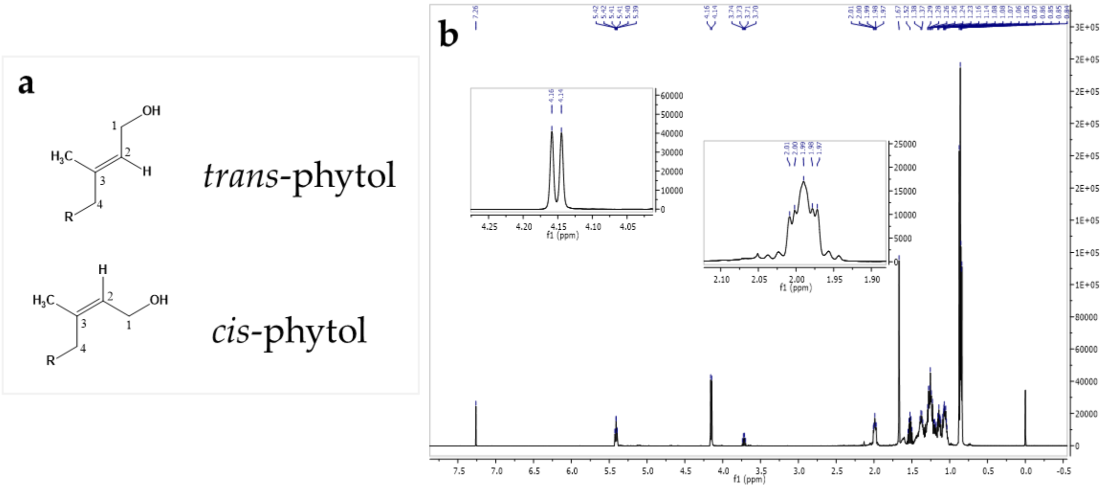

Figure S1. ^{1}H NMR analysis of phytol standard. ^{1}H NMR analysis of POH purchased from ARC® (b). The figure also shows the chemical formula of *cis*/*trans* isoprene (a) with the chemical groups commented in the text.

S2. RP-HPLC analysis of phytol. The non-radiolabelled POH standard and the radiolabeled POH were analyzed by RP-HPLC as described. Non-radiolabelled POH was monitored by UV detection (figure S2 A) and radiolabelled POH by scintillation counting of the collected fractions (figure S2 B). The results showed that both standards had identical peaks (figure S2) and no GGOH was detected (elution time ~9 min; data not shown).

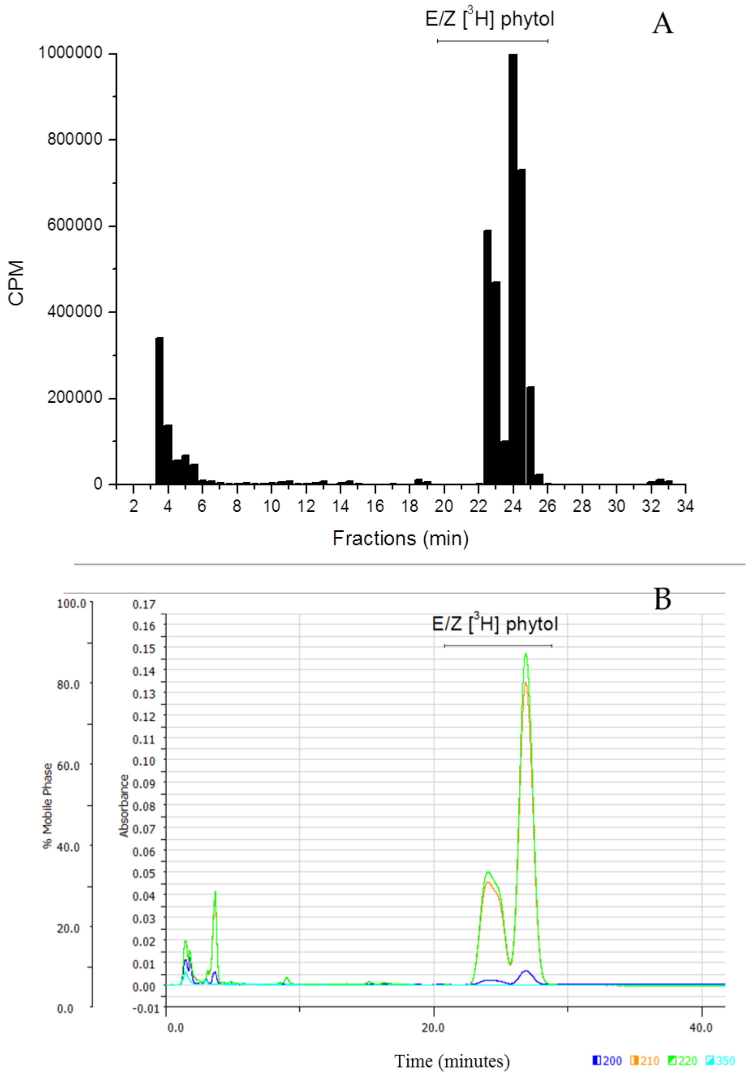

Figure S2. RP-HPLC analysis of phytol. RP-HPLC analysis of [1-(n)-^{3}H]-POH (B, up) and non-radiolabelled POH (A, down). Chromatographic analysis and radioactivity monitoring was performed as described in the methodological section. CPM counts per minute.

S3. Phytol and dolichols toxicity assays. The commercial mixture of dolichols 13-21 and POH was screened for their toxicity in malaria parasites. POH toxic effects for the parasite were only observed at concentrations >60 µM, and its IC_{50} value was estimated to be around 160 µM (figure S3 A). A mixture of dolichols showed to be toxic at concentrations up to 0.9 µg/ml (figure S3 B). No effects due to the vehicle were observed in any assay.

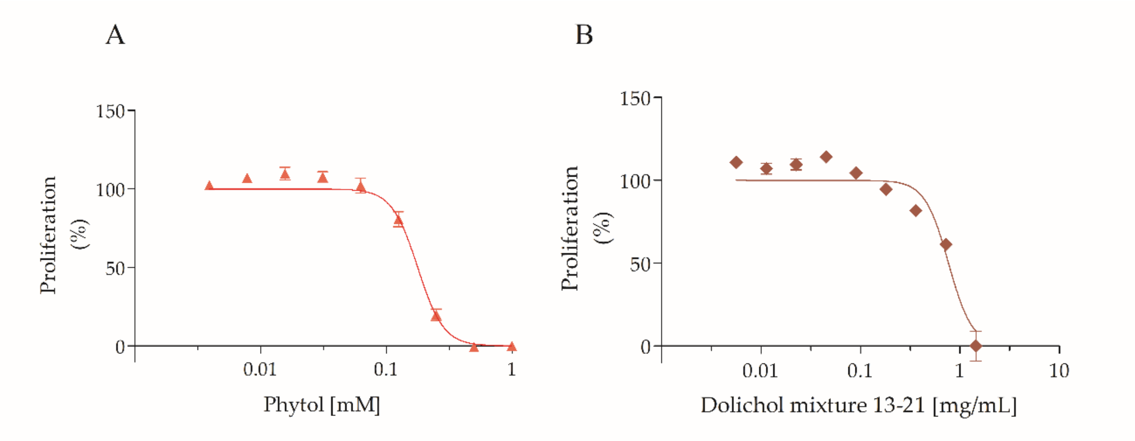

Figure S3. Phytol and dolichols toxicity. The figure shows the dose-response curve of POH and the commercial mixture of dolichols 13-21.

S4. Failed drug-rescue assays. The dose-response curve and IC_{50} value of fosmidomycin were calculated in the absence or presence of 5 µM of solanesol or 0.9 µg/ml of dolichols 13-21 in the culture medium (figure S4). After several attempts, we did not find any rescue phenomenon due to the supplementation with these isoprenoid alcohols.

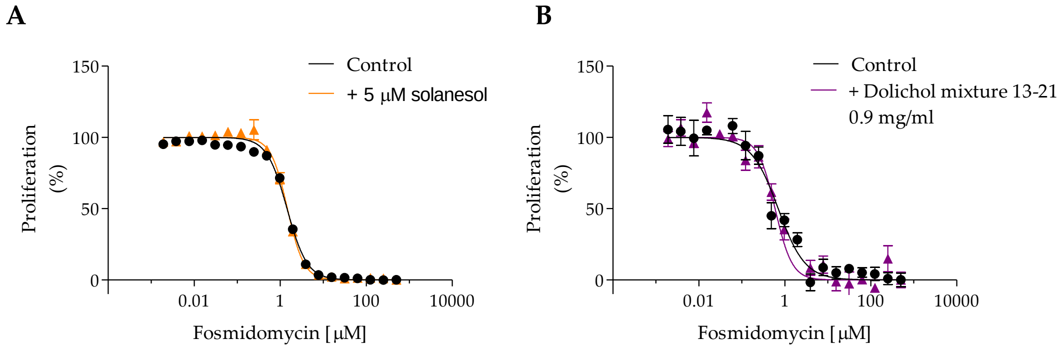

Figure S4. Failed drug-rescue assays. The figure shows the dose-response effect of the fosmidomycin and its IC_{50} in *P. falciparum* proliferation while adding (A) 5 µM of solanesol or (B) 0.9 mg/ml of dolichols 13-21 to the culture medium. After several attempts, we did not find any rescue phenomenon due to the supplementation. No effects due to the vehicle were observed in any assay. The figure shows one representative figure of three independent experiments with similar results.

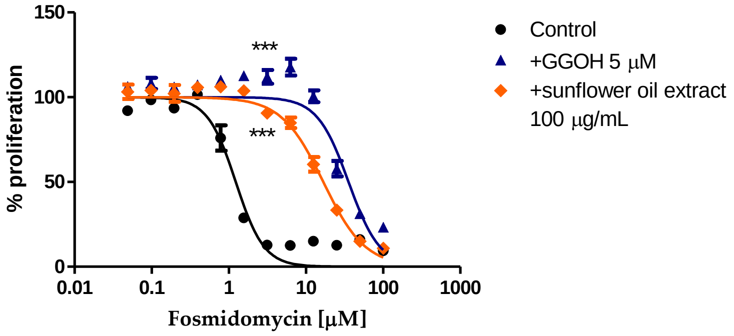

Figure S5. Fosmidomycin-rescue assays using sunflower oil and arugula extracts. (A) It was studied the fosmidomycin (initial concentration 100 µM) dose-response effect at 48 h in the presence or absence of 0.1 mg/ml sunflower oil and arugula extracts. Fosmidomycin IC_{50} = 0.96 ± 0.24 µM. Values represent the mean ± SD from three independent assays. These experiments were carried out using the SYTO11^{TM} staining methodology. Statistical analysis was performed with One Way ANOVA, followed by the Tukey posttest (*** $p < 0.005$ compared to the control)
